# Neurognathostomiasis, a Neglected Parasitosis of the Central Nervous System

**DOI:** 10.3201/eid1707.101433

**Published:** 2011-07

**Authors:** Juri Katchanov, Kittisak Sawanyawisuth, Verajit Chotmongkol, Yukifumi Nawa

**Affiliations:** Author affiliations: Mahidol University, Bangkok, Thailand (J. Katchanov, Y. Nawa);; Khon Kaen University, Khon Kaen, Thailand (K. Sawanyawisuth, V. Chotmongkol)

## MEDSCAPE CME

Medscape, LLC is pleased to provide online continuing medical education (CME) for this journal article, allowing clinicians the opportunity to earn CME credit.

This activity has been planned and implemented in accordance with the Essential Areas and policies of the Accreditation Council for Continuing Medical Education through the joint sponsorship of Medscape, LLC and Emerging Infectious Diseases. Medscape, LLC is accredited by the ACCME to provide continuing medical education for physicians.

Medscape, LLC designates this Journal-based CME activity for a maximum of 1 *AMA PRA Category 1 Credit(s)*^TM^. Physicians should claim only the credit commensurate with the extent of their participation in the activity.

All other clinicians completing this activity will be issued a certificate of participation. To participate in this journal CME activity: (1) review the learning objectives and author disclosures; (2) study the education content; (3) take the post-test and/or complete the evaluation at www.medscape.org/journal/eid; (4) view/print certificate.

**Release date: June 27, 2011; Expiration date: June 27, 2012**

## Learning Objectives

Upon completion of this activity, participants will be able to:

- Describe food sources of gnathostomiasis parasitic infection
- Describe different clinical manifestations of neurognathostomiasis infection
- Identify methods of diagnosis of neurognathostomiasis
- Describe treatment options for gnathostomiasis
- Describe outcomes of gnathostomiasis infection

## Editor

**Nancy Mannikko, **Technical Writer/Editor,* Emerging Infectious Diseases. Disclosure: Nancy Mannikko has disclosed no relevant financial relationships.*

## CME AUTHOR

**Desiree Lie, MD, MSEd, **Clinical Professor; Director of Research and Faculty Development, Department of Family Medicine, University of California Irvine at Orange. *Disclosure: Désirée Lie, MD, MSEd, has disclosed the following relevant financial relationship: served as a nonproduct speaker for "Topics in Health" for Merck Speaker Services.*

## AUTHORS

*Disclosures:* ***Juri Katchanov, MD; Kittisak Sawanyawisuth, MD; Verajit Chotmongkol, MD;*** *and* ***Yukifumi Nawa, MD,*** *have disclosed no relevant financial relationships.*
