# Supplementary material for: Neurognathostomiasis, a Neglected Parasitosis of the Central Nervous System
Source: Emerg Infect Dis. 2011 Jul;17(7):1174–80. doi: 10.3201/eid1707.101433 (PMC3321562; doi:10.3201/eid1707.101433)
Supplement: Related texts

# Review article: Neurognathostomiasis, a Neglected Parasitosis of the Central Nervous System

**DOI:** 10.3201/eid1707.101433

**Authors:** Juri Katchanov, Kittisak Sawanyawisuth, Verajit Chotmongkol, Yukifumi Nawa

**Affiliations:** Author affiliations: Mahidol University, Bangkok, Thailand (J. Katchanov, Y. Nawa);; Khon Kaen University, Khon Kaen, Thailand (K. Sawanyawisuth, V. Chotmongkol)

## Abstract

Gnathostomiasis is a foodborne zoonotic helminthic infection caused by the third-stage larvae of *Gnathostoma* spp. nematodes. The most severe manifestation involves infection of the central nervous system, neurognathostomiasis. Although gnathostomiasis is endemic to Asia and Latin America, almost all neurognathostomiasis cases are reported from Thailand. Despite high rates of illness and death, neurognathostomiasis has received less attention than the more common cutaneous form of gnathostomiasis, possibly because of the apparent geographic confinement of the neurologic infection to 1 country. Recently, however, the disease has been reported in returned travelers in Europe. We reviewed the English-language literature on neurognathostomiasis and analyzed epidemiology and geographic distribution, mode of central nervous system invasion, pathophysiology, clinical features, neuroimaging data, and treatment options. On the basis of epidemiologic data, clinical signs, neuroimaging, and laboratory findings, we propose diagnostic criteria for neurognathostomiasis.

Foodborne parasitic infections are common in the tropics, where many foodborne parasites are endemic and ingestion of raw shellfish and freshwater fish, as well as undercooked meat, is frequent among local populations (*1*). Increased international travel to areas endemic for these foodborne parasites and migration from tropical areas have led to the emergence of these diseases in temperate climates (*2*), where such infections are rarely seen by physicians and thus may not be considered in differential diagnoses. Gnathostomiasis is a foodborne zoonotic helminthic infection caused by the third-stage larva of *Gnathostoma* spp. nematodes (Figure 1, panels A, B). At least 13 species have been identified (*3*), with 5 recorded in humans. *G. spinigerum* is the most common of these nematodes in Asia. Human infection with *G. hispidum*, *G. doloresi*, and *G. nipponicum* were found only in Japan (*4*). In the Americas, *G. binucleatum* is the only proven pathogenic *Gnathostoma* nematode in humans. Humans are infected primarily by eating raw or undercooked freshwater fish (Figure 1, panel C), frogs, and chicken. Humans are accidental unsuitable hosts; the parasite rarely develops to an adult worm, and the disease in humans is caused by the migrating larva.

**Figure 1:** A) Third-stage larva of the nematode *Gnathostoma* sp. Scale bar = 250 µm. B) Scanning electronic microscopy image depicting head bulb with 4 cephalic hooklet rows. Original magnification ×500. C) *Gnathostoma* sp. larvae in the flesh of their intermediate host, *Eleotris picta* fish. Original magnification ×4. Inset: Higher magnification of an encysted larva; original magnification ×100. Larvae photographs courtesy of Dr Diaz-Camacho, Universidad Autónoma de Sinaloa, Sinaloa, Mexico. D) Cross section of a *Gnathostoma* sp. larva in human skin biopsy sample (hematoxylin and eosin stain). Scale bar = 250 µm.

Gnathostomiasis can be divided into cutaneous, visceral, and ocular forms, depending on the site of larval migration and subsequent signs and symptoms (*2*). The most common clinical presentation is the cutaneous one (Figure 1, panel D), which is characterized by localized, intermittent, migratory swellings of the skin and is often associated with localized pain, pruritus, and erythema (*5**,**6*). Visceral involvement can manifest in virtually any organ and any part of the body (*3*). The most severe manifestation of the visceral disease is involvement of the central nervous system (CNS), i.e., neurognathostomiasis. Neurognathostomiasis has been reported only in *G. spinigerum* infections (*3*).

We found 24 reports describing a total of 248 patients with neurognathostomiasis published in English-language literature. In this article, we review epidemiology, mode of CNS invasion, pathophysiology, clinical features, neuroimaging data, and treatment options, and we propose diagnostic criteria for this emerging disease.

## History of Neurognathostomiasis

The first clinical case of *G. spinigerum* meningoencephalitis was reported by Daensvang in 1949 (*7*). Eighteen years later, Chitanondh and Rosen (*8*) documented the first pathologic evidence of *G. spinigerum* invasion of the CNS from the autopsy of a 34-year-old Thai woman. In their seminal study, Boongird et al. (*9*) delineated the clinical syndromes of neurognathostomiasis, which facilitated further prospective and retrospective case identification (*10*). With the advance of high-resolution neuroimaging, the parasitic tracks of *Gnathostoma* could be demonstrated on magnetic resonance imaging (MRI), aiding the diagnosis of neurognathostomiasis in the absence of worm recovery (*11*).

## Epidemiology of Neurognathostomiasis

Most (241/248) identified cases of neurognathostomiasis reported in the English-language literature were from Thailand. Two patients were infected in Laos (*12**,**13*), 1 in Japan (*14*), 1 in Myanmar (*13*), and 2 in South Korea (*15**,**16*). One patient had traveled to Southeast Asia and Japan (*17*), so the geographic origin of his infection could not be determined. The incubation period of neurognathostomiasis can be as long as 10 years because of the long persistence of the nematode larvae in the soft tissues before CNS invasion (*2**,**15*), which means it can occur in immigrants in a country in which the parasite is not endemic (*16**,**18*). All case-patients were reported to be infected with *G. spinigerum*. Although gnathostomiasis has been increasingly detected outside of Asia, particularly in Mexico, no cases of neurognathostomiasis have been reported in other regions. The occurrence of another *Gnathostoma* species in Latin America, namely *G. binucleatum*, is a possible explanation for this phenomenon.

## Neurognathostomiasis in Travelers

The first case of neurognathostomiasis in a traveler was reported in 2003 (*19*). Since then, 4 patients in Europe returning from gnathostomiasis-endemic countries have been published (*13**,**20**,**21*). Two patients traveled to Thailand (*19**,**20*), 1 patient to Lao People’s Democratic Republic (*13*) and 1 patient to Myanmar (*13*). The patients were reported to have indulged in local customs of eating raw fish (*19*) and shrimp (*20*). Gnathostomiasis has been recently reported in travelers returning from southern Africa (*22**,**23*). However, the patients had cutaneous manifestation of *Gnathostoma* spp. infection without neurologic involvement.

## Pathophysiology of Neurognathostomiasis

*Gnathostoma* spp. nematodes are highly invasive parasites. After being ingested, the third-stage larvae penetrate the mucosal wall of the gastrointestinal tract. Larvae can enter the CNS by invading directly through the loose connective tissues of the neural foramina of the skull base along the cranial nerves and vessels or through intervertebral foramina along the spinal nerves and vessels (*24*). The larvae release a variety of molecules into their surrounding environment that facilitate tissue penetration and invasion (*25*). Proteases are the major molecular types among various components of these so-called excretor–secretory products, both quantitatively and qualitatively (*26*). In *Gnathostoma* spp., matrix metalloproteinases might play a key role in invasion of host tissues (*27*).

*Gnathostoma* spp. nematodes typically enter the spinal cord along the nerve roots, causing radiculomyelitis (*9**,**28*). The parasite is then able to ascend the spinal cord and reach the brain (*3**,**29*). This journey may take as long as several years and has been documented in a Thai patient through sequential imaging (*29*).

Given the length of the migrating larva, which averages 3.0 mm (*30*), the migration of the worm in the CNS causes direct mechanical injury because of tearing of nerve tissue. The hallmark sign of neurognathostomiasis is the evidence of multiple hemorrhagic tracks; these tracks have been documented throughout the spinal cord and brain tissue by postmortem examination (*9*), as well as through neuroimaging (*31*). Subarachnoid hemorrhage can result from larvae burrowing through a cerebral arteriole (*32**,**33*). Eosinophilic meningitis caused by *Gnathostoma* spp. is characterized by erythrocytes in cerebrospinal fluid (CSF) (*33*), which suggests mechanical damage by the worm. Apart from the structural damage, the inflammatory response to the migrating larvae might further contribute to tissue destruction (*34*).

## Clinical Signs of Neurologic Involvement

The main clinical syndromes of neurognathostomiasis are radiculomyelitis/ radiculomyeloencephalitis, meningitis/meningoencephalitis, and subarachnoid and intracerebral hemorrhage (Table 1). Each syndrome reflects the tissue injury by the migrating parasite. Because the larvae migrate, the same patient can show sequentially different neurologic syndromes. For example, in 1 Laotian patient, meningomyelitis developed, followed by subarachnoid hemorrhage (*12*). Meningitis, subdural hemorrhage, and then intraparenchymal lesions developed sequentially in 1 patient from northeastern Thailand (*29*). The most common symptom reported virtually by every patient was pain: radicular pain in cases of spinal involvement (often lasting 1–5 days) or headache in cases of meningoencephalitis. The most common manifestation, spinal cord disease, comprised 55% of all clinical syndromes (Table 1).

**Table 1:** Clinical presentation of 248 patients with neurognathostomiasis*

Syndrome	Probable entry portal entry	Clinical signs and symptoms	No. (%) cases
Radiculomyelitis/myelitis/ myeloencephalitis	Intervertebral foramina along the spinal nerves and vessels	Sharp radicular pain and a spinal syndrome (paraplegia, monoplegia, quadriplegia, bladder dysfunction, sensory disturbances), can progress to cerebral involvement (myeloencephalitis)	140 (55)
Meningitis/ meningoencephalitis	Neural foramina of the skull base along the cranial nerves and vessels	Severe headache, stiffness of the neck, cranial nerve palsies, disturbance of consciousness, focal neurologic signs	77 (30)
Intracerebral hemorrhage	Intervertebral or neural foramina	Headache, sudden-onset focal neurologic signs	21 (8)
Subarachnoid hemorrhage	Intervertebral or neural foramina	Thunderclap headache, meningeal signs	16 (7)
*Because the larvae migrate, patients can have sequential signs and symptoms; thus, the total number of clinical syndromes shown exceeds the number of reported patients.			

Myelopathy in gnathostomiasis is characterized by radicular pain followed by an ascending paresis of lower extremities or quadriparesis with bladder dysfunction (*9**,**10**,**33**,**35**–**38*). A sensory level for all modalities including vibration and proprioception was a frequent finding, commonly localized in the thoracic region (*9*). Cerebral and meningeal involvement is characterized by meningeal signs and decreased consciousness. Abducens nerve palsy was the most common cranial nerve impairment (*9**,**33*). Intracranial hemorrhages were seen with sudden-onset focal neurologic deficits such as hemiparesis and hemihypaesthesia (*9**,**10**,**18**,**33*).

### Isolated Neurologic Disease

The most common clinical feature of gnathostomiasis is intermittent migratory cutaneous swelling (*5*). However, in the largest published series of neurognathostomiasis in 162 patients, only 11 (7%) had a history of cutaneous lesions (*10*). Unilateral eyelid swelling, another typical sign of gnathostomiasis, occurred in only 6 patients (4%) (*10*). Boongird et al. reported only 3 patients with subcutaneous swelling out of 24 patients with neurognathostomiasis; cutaneous gnathostomiasis developed in all of these patients after the onset of neurologic symptoms (*9*).

### CSF Studies

Eosinophilic pleocytosis is a hallmark laboratory finding. CSF eosinophilia in patients is usually prominent; median values were 40% (n = 24) and 54% (n = 39) in the studies by Boongird et al (*9*) and Schmutzhard et al (*33*), respectively. In 109 (67%) of patients reported by Punyagupta et al. (*10*), the eosinophil count in CSF was >30%. CSF has been reported as xanthochromic or bloody in 134 patients (64% of all published cases). CSF glucose is usually only mildly reduced but was reported as low as 11 mg/dL (9% of the plasma value) in 1 patient (*37*).

### Neuroradiologic Features

Neuroimaging of 11 patients with neurognathostomiasis was reported in English-language literature. On cranial computed tomography, subarachnoid hemorrhage and intracerebral hemorrhage were the most common findings (*12**,**18**,**33*). In 1 patient a subdural hemorrhage was detected (*29*). The hallmark of cerebral gnathostomiasis on MRI was the detection of hemorrhagic tracks (Figure 2, panels A, C, D). The magnetic resonance signal of a hemorrhagic lesion varies in acute, subacute, and chronic lesions. Hence, on T1 and T2 weighted imaging both hypointensities and hyperintensities have been documented. No gradient-echo T2-weighted sequences have been reported in neurognathostomiasis. However, gradient-echo T2-weighted sequences are exquisitely sensitive to local field inhomogeneities of hematoma and could become a useful sequence in radiologic assessment of cerebral gnathostomiasis.

**Figure 2:** Images of the brains of patients with cerebral gnathostomiasis. A) Axial T1-weighted image showing small hemorrhage in the right basal ganglia (arrow). B) Sagittal T2-weighted images showing diffuse cord enlargement with longitudinal T2 hyperintensity (arrows). C) Axial T1-weighted image showing a hemorrhagic track in the tegmentum of the pons (arrow). D) Coronal T1-weighted postgadolinium image, showing the longitudinal extension of the same hemorrhagic track as in panel C (arrows). Images from K. Sawanyawisuth et al. (*11*), used with permission.

Diffuse multisegmental spinal cord swelling with corresponding T2 hyperintensities was the most common finding on spinal imaging (*11**,**17*) (Figure 2, panel B). Gadolinium enhancement of the lesions was reported in cranial and spinal MRI of 4 patients as slight, nodular, and ill-defined (*11**,**17**,**35*). Table 2 summarizes typical findings on neuroimaging in neurognathostomiasis.

**Table 2:** Neuroradiologic features of neurognathostomiasis*

Site	Procedure	Findings
Brain	CT	Parenchymal (single or multiple), subdural or subarachnoid hyperdensities corresponding to intracranial hemorrhage
	MRI	Multiple (worm-like) T2-weighted hyperintensities or hypointensities in both hemispheres and the cerebellum of >3 mm diameter (hemorrhagic tracks) with or without gadolinium enhancement
Spinal cord	MRI	Dilatation/swelling of the spinal cord with multisegmental T2-weighted hyperintensities, frequent gadolinium enhancement (slight to moderate) on T1 postcontrast images

*CT, computed tomography; MRI, magnetic resonance imaging.

### Immunodiagnosis

The detection of antibodies in serum is the main pillar of immunodiagnosis of gnathostomiasis. Two methods have been established for clinical routine: ELISA and Western blot by using crude *Gnathostoma* spp. antigens from the larval extract. The 24-kD band on Western blot was shown to have nearly 100% specificity for gnathostomiasis (*39*). The current practice for serologic diagnosis is to use the ELISA (e.g., multiple-dot ELISA) as the first step (*40*) and to confirm the results by Western blot. At least 2 patients seroconverted during their neurologic illness (*19**,**20*), which emphasizes the need for repeated serologic examination in cases with strong clinical suspicion. The serologic testing can be done in several laboratories, including those in Thailand (Bangkok, Khon Kaen), Japan (Miayzaki), and Europe (Basel, Switzerland). Additional information about laboratories providing this service is available from the corresponding author.

### Differential Diagnosis

The main differential diagnosis of neurognathostomiasis is an infection with *Angiostrongylus cantonensis*. However, *Angiostrongylus* spp. infection is usually seen as self-limited eosinophilic meningitis and only rarely causes severe disease with prominent spinal or cerebral involvement (*1*). Immunodiagnostically, positive 24-kD band against *G. spinigerum* antigen and negative 29-kD and 31-kD bands on immunoblot against *A. cantonensis* antigen make a diagnosis of an *Angiostrongylus* spp. infection highly unlikely (*40*). Other causes of a parasitic CNS disease, such as cysticercosis, toxocariasis, schistosomiasis, baylisascariasis, or paragonimiasis, can be distinguished by epidemiologic distribution, clinical signs and symptoms, radiologic appearance, and serologic testing.

## Treatment

No randomized trials of antihelminthic therapy have been conducted for neurognathostomiasis. We have identified 9 reports of treatment of neurognathostomiasis with albendazole (*11**,**13**,**17**–**21**,**29*); 5 patients fully recovered, 2 partially recovered, and 2 patients did not recover. Outcomes might reflect the natural course of the disease rather than the effect of treatment. In several reports, antihelminthic treatment was considered as potentially detrimental and was withheld on the basis of its possible induction of brain edema due to sudden helminthic death (*31**,**38*). Three returned travelers from Thailand were treated with albendazole (800 mg/d for 1 month [*19*], 800 mg/d for 3 weeks [*20*], and 400 mg 2×/d for 4 weeks [*21*]) and fully recovered (*19**–**21*). In all these patients, dexamethasone (oral or intravenous) or predinsolone was added to the treatment regimen. A recent retrospective evaluation of long-term treatment efficacy in 13 patients from France with imported gnathostomiasis reported 2 patients with neurologic involvement (*13*), 1 with myelitis, and 1 with encephalitis (C. Strady, pers. comm.). The patient with myelitis fully recovered after albendazole therapy, and the patient with encephalitis was reported to have had 4 relapses despite therapy with ivermectin and albendazole (*13*; C. Strady, pers. comm.).

Corticosteroids have been used in neurognathostomiasis to treat cerebral and spinal edema (*19**–**21*). They also might prevent or alleviate paradoxical worsening after initiation of antihelminthic treatment. However, no conclusion about their efficacy can be drawn from the current data because no randomized control trials have been conducted and the retrospective data are too limited to allow any comparison between the groups. At the moment, no evidence-based recommendations can be issued.

### Outcome

The case-fatality rate in the first series of 24 patients was 25% (*9*). Later studies reported mortality rates of 7%–12% (*10**,**33*). However, as the authors remark, these case-fatality rates are almost certainly an underestimate because many patients in critical condition were discharged home to die (*10*). In total, the outcome of 247 patients has been reported in the literature. An unfavorable outcome, e.g., death or severe persistent disability, was reported in 78 patients (32%). However, the recent case reports indicate that the current prognosis might be better. Of 15 patients in whom neurognathostomiasis was diagnosed after 2001, poor outcomes were reported in 3 (20%) patients. In all 4 clinical cases of returned travelers with myelitis, recovery was good after antihelminthic therapy (*13**,**19**–**21*).

## Conclusions

Definitive diagnosis of helminthic CNS infections is notoriously difficult. The recovery rate of worms from the CSF is low, and the invasive procedures for diagnosis are seldom justified (*33**,**35*). Neurognathostomiasis is no exception; of 248 published cases, only 27 (11%) were confirmed by recovery of larvae. Moreover, an intermittent subcutaneous swelling, the hallmark of gnathostomiasis, is found only in the minority of neurognathostomiasis cases. Given these difficulties, clinical criteria are useful for practice and research. On the basis of the analysis of published cases, we propose diagnostic criteria that should guide a clinician if a pathologic diagnosis is not possible (Table 3).

**Table 3:** Proposed diagnostic criteria for neurognathostomiasis*

Epidemiologic criteria
Travel or residence in the disease-endemic area AND exposure to undercooked freshwater fish, frogs, poultry, and shellfish
Clinical syndrome
Painful radiculomyelitis/radiculomyeloencephalitis OR meningitis/meningoencephalitis OR intracerebral hemorrhage OR subarachnoid hemorrhage
Indirect evidence of CNS invasion
CSF studies: eosinophilic pleocytosis OR neuroimaging: parenchymal hemorrhagic tracks (>3 mm)
Immunodiagnosis
Positive detection of reactivity against the 24-kDa component of *Gnathostoma spinigerum* in immunoblot test†
Absence of reactivity against the 29–31-kDa components of *Angiostrongylus cantonensis* in immunoblot test

*All criteria should be fullfiled for clinical diagnosis of neurognathostomiasis. CNS, central nervous system; CSF, cerebrospinal fluid. †If negative, a follow-up examination 4 weeks later is recommended.

Neurognathostomiasis is a parasitic CNS disease endemic to Southeast Asia, particularly Thailand. Because of growing international travel to disease-endemic areas, as well as migration from the tropics, an increasing number of patients will seek medical attention in Europe and North America. Neurognathostomiasis should be suspected in patients who have eosinophilic radiculomyelitis, myeloencephalitis or meningoencephalitis, and a history of travel to gnathostomiasis-endemic areas. Ingestion of raw or inadequately cooked shellfish, freshwater fish, or poultry is a prerequisite for infection; however, because such information might not be volunteered by the patient, clinicians should ask directly about foods eaten during travel. Serologic testing, including Western blot, shows high specificity and can contribute to the diagnosis in an appropriate clinical and epidemiologic setting. No evidence-based protocol is available for treatment of neurognathostomiasis; however, a 3- to 4-week course of albendazole with prednisolone or dexamethasone is 1 of the most frequently used regimens. Clinicians should advise patients traveling to or living in disease-endemic areas to avoid eating undercooked freshwater fish, frogs, poultry, and shellfish.

## References

[1] Nawa Y, Hatz C, Blum J. Sushi delights and parasites: the risk of fishborne and foodborne parasitic zoonoses in Asia. Clin Infect Dis. 2005;41:1297–303. 10.1086/49692016206105

[2] Moore DA, McCroddan J, Dekumyoy P, Chiodini PL. Gnathostomiasis: an emerging imported disease. Emerg Infect Dis. 2003;9:647–50.1278100310.3201/eid0906.020625PMC3000140

[3] Nawa Y. Historical review and current status of gnathostomiasis in Asia. Southeast Asian J Trop Med Public Health. 1991;22(Suppl):217–9.1822889

[4] Herman JS, Chiodini PL. Gnathostomiasis, another emerging imported disease. Clin Microbiol Rev. 2009;22:484–92. 10.1128/CMR.00003-0919597010PMC2708391

[5] Rusnak JM, Lucey DR. Clinical gnathostomiasis: case report and review of the English-language literature. Clin Infect Dis. 1993;16:33–50. 10.1093/clinids/16.1.338448317

[6] Chai JY, Han ET, Shin EH, Park JH, Chu JP, Hirota M, An outbreak of gnathostomiasis among Korean emigrants in Myanmar. Am J Trop Med Hyg. 2003;69:67–73.12932100

[7] Daengsvang S. Human gnathostomiasis in Siam with reference to the method of prevention. J Parasitol. 1949;35:116–21. 10.2307/327311218119157

[8] Chitanondh H, Rosen L. Fatal eosinophilic encephalomyelitis caused by the nematode *Gnathostoma spinigerum.* Am J Trop Med Hyg. 1967;16:638–45.605353110.4269/ajtmh.1967.16.638

[9] Boongird P, Phuapradit P, Siridej N, Chirachariyavej T, Chuahirun S, Vejjajiva A. Neurological manifestations of gnathostomiasis. J Neurol Sci. 1977;31:279–91. 10.1016/0022-510X(77)90113-7839236

[10] Punyagupta S, Bunnag T, Juttijudata P. Eosinophilic meningitis in Thailand. Clinical and epidemiological characteristics of 162 patients with myeloencephalitis probably caused by *Gnathostoma spinigerum.* J Neurol Sci. 1990;96:241–56. 10.1016/0022-510X(90)90136-B2376755

[11] Sawanyawisuth K, Tiamkao S, Kanpittaya J, Dekumyoy P, Jitpimolmard S. MR imaging findings in cerebrospinal gnathostomiasis. AJNR Am J Neuroradiol. 2004;25:446–9.15037471PMC8158538

[12] Brant-Zawadzki M, Wofsy CB, Schechter G. CT-evidence of subarachnoid hemorrhage due to presumed gnathostomiasis. West J Med. 1982;137:65–7.7135942PMC1273996

[13] Strady C, Dekumyoy P, Clement-Rigolet M, Danis M, Bricaire F, Caumes E. Long-term follow-up of imported gnathostomiasis shows frequent treatment failure. Am J Trop Med Hyg. 2009;80:33–5.19141836

[14] Kawamura J, Kohri Y. Nobuyuki 0. Eosinophilic meningoradiculo-myelitis caused by *Gnathostoma spinigerum*: a case report. Arch Neurol. 1983;40:583–5.661529110.1001/archneur.1983.04050080083017

[15] Lo Re V III, Gluckman SJ. Eosinophilic meningitis due to *Gnathostoma spinigerum.* J Infect. 2002;45:117–20. 10.1053/jinf.2002.101012217718

[16] Catalano M, Kaswan D, Levi MH. Wider range for parasites that cause eosinophilic meningitis. Clin Infect Dis. 2009;49:1283. 10.1086/60568719780663

[17] Chandenier J, Husson J, Canaple S, Gondry-Jouet C, Dekumyoy P, Danis M, Medullary gnathostomiasis in a white patient: use of immunodiagnosis and magnetic resonance imaging. Clin Infect Dis. 2001;32:E154–7. 10.1086/32052011340549

[18] Germann R, Schächtele M, Nessler G, Seitz U, Kniehl E. Cerebral gnathostomiasis as a cause of an extended intracranial bleeding. Klin Padiatr. 2003;215:223–5. 10.1055/s-2003-4140112929012

[19] Górgolas M, Santos-O'Connor F, Unzú AL, Fernández-Guerrero ML, Gárate T, Troyas Guarch RM, Cutaneous and medullar gnathostomiasis in travelers to Mexico and Thailand. J Travel Med. 2003;10:358–61. 10.2310/7060.2003.937314642206

[20] Elzi L, Decker M, Battegay M, Rutishauser J, Blum J. Chest pain after travel to the tropics. Lancet. 2004;363:1198. 10.1016/S0140-6736(04)15950-315081653

[21] Schmutzhard E. Eosinophilic myelitis, a souvenir from South East Asia. Pract Neurol. 2007;7:48–51.17430865

[22] Herman JS, Wall EC, van-Tulleken C, Godfrey-Faussett P, Bailey RL, Chiodini PL. Gnathostomiasis acquired by British tourists in Botswana. Emerg Infect Dis. 2009;15:594–7. 10.3201/eid1504.08164619331741PMC2671435

[23] Hale DC, Blumberg L, Frean J. Case report: gnathostomiasis in two travelers to Zambia. Am J Trop Med Hyg. 2003;68:707–9.12887031

[24] Katchanov J, Nawa Y. Helminthic invasion of the central nervous system: many roads lead to Rome. Parasitol Int. 2010;59:491–6. 10.1016/j.parint.2010.08.00220709186

[25] Maruyama H, Nawa Y. Immunology of the infection. In: Murrell KD, Fried B, editors. Worldclass parasites, vol. 11, food-borne parasitic zoonoses. Fish and plant-borne parasites. New York: Springer; 2007. p. 337–81.

[26] Tort J, Brindley PJ, Knox D, Wolfe KH, Dalton JP. Proteinases and associated genes of parasitic helminths. Adv Parasitol. 1999;43:161–266. 10.1016/S0065-308X(08)60243-210214692

[27] Uparanukraw P, Morakote N, Harnnoi T, Dantrakool A. Molecular cloning of a gene encoding matrix metalloproteinase-like protein from *Gnathostoma spinigerum.* Parasitol Res. 2001;87:751–7. 10.1007/s00436010044011570561

[28] Punyagupta S, Limtrakul C, Vichipanthu P, Karnchanachetanee C, Nye SW. Radiculomyeloencephalitis associated with eosinophilic pleocytosis—report of nine cases. Am J Trop Med Hyg. 1968;17:551–60.567278410.4269/ajtmh.1968.17.551

[29] Sawanyawisuth K, Chlebicki MP, Pratt E, Kanpittaya J, Intapan PM. Sequential imaging studies of cerebral gnathostomiasis with subdural hemorrhage as its complication. Trans R Soc Trop Med Hyg. 2009;103:102–4. 10.1016/j.trstmh.2008.09.01118951600

[30] Rojekittikhun W, Pubampen S. Morphological variation and abnormality of cephalic hooklets of *Gnathostoma spinigerum* hepatic stage larvae from laboratory infected mice. Southeast Asian J Trop Med Public Health. 1998;29:118–22.9740283

[31] Sithinamsuwan P, Chairangsaris P. Images in clinical medicine. Gnathostomiasis—neuroimaging of larval migration. N Engl J Med. 2005;353:188. 10.1056/NEJMicm04079516014888

[32] Visudhiphan P, Chiemchanya S, Somburanasin R, Dheandhanoo D. Causes of spontaneous subarachnoid hemorrhage in Thai infants and children. A study of 56 patients. J Neurosurg. 1980;53:185–7. 10.3171/jns.1980.53.2.01857431057

[33] Schmutzhard E, Boongird P, Vejjajiva A. Eosinophilic meningitis and radiculomyelitis in Thailand, caused by CNS invasion of *Gnathostoma spinigerum* and *Angiostrongylus cantonensis.* J Neurol Neurosurg Psychiatry. 1988;51:80–7. 10.1136/jnnp.51.1.803351533PMC1032717

[34] Punyagupta S, Juttijudata P, Bunnag T, Comer DS. Two fatal cases of eosinophilic myeloencephalitis a newly recognized disease caused by *Gnathostoma spinigerum.* Trans R Soc Trop Med Hyg. 1968;62:801–9. 10.1016/0035-9203(68)90008-45729570

[35] Bunyaratavej K, Pongpunlert W, Jongwutiwes S, Likitnukul S. Spinal gnathostomiasis resembling an intrinsic cord tumor/myelitis in a 4-year-old boy. Southeast Asian J Trop Med Public Health. 2008;39:800–3.19058570

[36] Bunnag T, Comer DS, Punyagupta S. Eosinophilic myeloencephalitis caused by *Gnathostoma spinigerum*. Neuropathology of nine cases. J Neurol Sci. 1970;10:419–34. 10.1016/0022-510X(70)90023-75444893

[37] Daengsvang S. A monograph on the genus *Gnasthostoma* and gnathostomiasis in Thailand. Tokyo: Southeast Asian Medical Information Center International Medical Foundation of Japan; 1980.

[38] Sawanyawisuth K, Tiamkao S, Nitinavakarn B, Dekumyoy P, Jitpimolmard S. MR imaging findings in cauda equina gnathostomiasis. AJNR Am J Neuroradiol. 2005;26:39–42.15661696PMC7975055

[39] Laummaunwai P, Sawanyawisuth K, Intapan PM, Chotmongkol V, Wongkham C, Maleewong W. Evaluation of human IgG class and subclass antibodies to a 24 kDa antigenic component of *Gnathostoma spinigerum* for the serodiagnosis of gnathostomiasis. Parasitol Res. 2007;101:703–8. 10.1007/s00436-007-0538-317468971

[40] Eamsobhana P, Ongrotchanakun J, Yookek A, Punthuprapasa P, Monkong N, Dekumyoy P. Multi-immunodot for rapid differential diagnosis of eosinophilic meningitis due to parasitic infections. J Helminthol. 2006;80:249–54.16923267

# Review article: Neurognathostomiasis, a Neglected Parasitosis of the Central Nervous System

**DOI:** 10.3201/eid1707.101433

**Authors:** Juri Katchanov, Kittisak Sawanyawisuth, Verajit Chotmongkol, Yukifumi Nawa

**Affiliations:** Author affiliations: Mahidol University, Bangkok, Thailand (J. Katchanov, Y. Nawa);; Khon Kaen University, Khon Kaen, Thailand (K. Sawanyawisuth, V. Chotmongkol)

## Earning CME Credit

To obtain credit, you should first read the journal article. After reading the article, you should be able to answer the following, related, multiple-choice questions. To complete the questions and earn continuing medical education (CME) credit, please go to www.medscape.org/journal/eid. Credit cannot be obtained for tests completed on paper, although you may use the worksheet below to keep a record of your answers. You must be a registered user on Medscape.org. If you are not registered on Medscape.org, please click on the New Users: Free Registration link on the left hand side of the website to register. Only one answer is correct for each question. Once you successfully answer all post-test questions you will be able to view and/or print your certificate. For questions regarding the content of this activity, contact the accredited provider, CME@medscape.net. For technical assistance, contact CME@webmd.net. American Medical Association’s Physician’s Recognition Award (AMA PRA) credits are accepted in the US as evidence of participation in CME activities. For further information on this award, please refer to http://www.ama-assn.org/ama/pub/category/2922.html. The AMA has determined that physicians not licensed in the US who participate in this CME activity are eligible for *AMA PRA Category 1 Credits*™. Through agreements that the AMA has made with agencies in some countries, AMA PRA credit is acceptable as evidence of participation in CME activities. If you are not licensed in the US and want to obtain an AMA PRA CME credit, please complete the questions online, print the certificate and present it to your national medical association.

## Article Title: Neurognathostomiasis, a Neglected Parasitosis of the Central Nervous System

## CME Questions

**1. A traveler returning from Thailand is suspected of having neurognathostomiasis. Which of the following is least likely to be a food source for the infection?**A. Undercooked porkB. Undercooked chickenC. Raw fishD. Undercooked frog**2. In the patient from question 1, which of the following is likely to be the most common presentation?**A. Intracerebral hemorrhage with headache and hemiparesisB. Myelopathy with radicular pain and ascending paresisC. Meningoencephalitis with photophobia and seizuresD. Subarachnoid hemorrhage with loss of consciousness**3. Which of the following is considered the most accurate test for the diagnosis of neurognathostomiasis?**A. Cerebrospinal fluid microscopyB. Brain biopsyC. Western blot serologyD. Spinal fluid culture**4. Which of the following treatments are believed to be most efficacious based on clinical trials?**A. Albendazole alone for 4 weeksB. Albendazole with prednisoloneC. Ivermectin with albendazoleD. None of the above**5. Which of the following best describes the most recent reports (after 2000) of rates of poor outcomes associated with gnathostomiasis infection?**A. Over 50% mortalityB. Mortality or severe disability rate of 40%C. Poor outcome in 30%D. Poor outcome in 20%

## Activity Evaluation

**Table:** 

1. The activity supported the learning objectives.				
Strongly Disagree				Strongly Agree
1	2	3	4	5
2. The material was organized clearly for learning to occur.				
Strongly Disagree				Strongly Agree
1	2	3	4	5
3. The content learned from this activity will impact my practice.				
Strongly Disagree				Strongly Agree
1	2	3	4	5
4. The activity was presented objectively and free of commercial bias.				
Strongly Disagree				Strongly Agree
1	2	3	4	5
